# The Search for Biomarkers and Treatments in Chagas Disease: Insights From TGF-Beta Studies and Immunogenetics

**DOI:** 10.3389/fcimb.2021.767576

**Published:** 2022-02-02

**Authors:** Roberto Rodrigues Ferreira, Mariana Caldas Waghabi, Sabine Bailly, Jean-Jacques Feige, Alejandro M. Hasslocher-Moreno, Roberto M. Saraiva, Tania C. Araujo-Jorge

**Affiliations:** ^1^ Laboratory of Innovations in Therapies, Education and Bioproducts, Oswaldo Cruz Institute (LITEB-IOC/Fiocruz), Oswaldo Cruz Foundation (Fiocruz), Rio de Janeiro, Brazil; ^2^ Laboratory of Functional Genomics and Bioinformatics, Oswaldo Cruz Institute (LAGFB-IOC/Fiocruz), Oswaldo Cruz Foundation (Fiocruz), Rio de Janeiro, Brazil; ^3^ Laboratory Biology of Cancer and Infection, Université Grenoble Alpes, Inserm, Commissariat à l’Energie Atomique, Grenoble, France; ^4^ Clinical Research Laboratory of Chagas Disease, Evandro Chagas National Institute of Infectious Disease, Oswaldo Cruz Foundation, Rio de Janeiro, Brazil

**Keywords:** Chagas disease, TGF-beta, fibrosis, biomarker, polymorphism

## Abstract

The anti-inflammatory cytokine transforming growth factor beta (TGF-β) plays an important role in Chagas disease (CD), a potentially life-threatening illness caused by *Trypanosoma cruzi*. In this review we revisited clinical studies in CD patients combined with *in vitro* and *in vivo* experiments, presenting three main sections: an overview of epidemiological, economic, and clinical aspects of CD and the need for new biomarkers and treatment; a brief panorama of TGF-β roles and its intracellular signaling pathways, and an update of what is known about TGF-β and Chagas disease. In *in vitro* assays, TGF-β increases during *T. cruzi* infection and modulates heart cells invasion by the parasite fostering its intracellular parasite cycle. TGF-β modulates host immune response and inflammation, increases heart fibrosis, stimulates remodeling, and slows heart conduction *via* gap junction modulation. TGF-β signaling inhibitors reverts these effects opening a promising therapeutic approach in pre-clinical studies. CD patients with higher TGF-β1 serum level show a worse clinical outcome, implicating a predictive value of serum TGF-β as a surrogate biomarker of clinical relevance. Moreover, pre-clinical studies in chronic *T. cruzi* infected mice proved that inhibition of TGF-β pathway improved several cardiac electric parameters, reversed the loss of connexin-43 enriched intercellular plaques, reduced fibrosis of the cardiac tissue, restored GATA-6 and Tbox-5 transcription, supporting cardiac recovery. Finally, TGF-β polymorphisms indicate that CD immunogenetics is at the base of this phenomenon. We searched in a Brazilian population five single-nucleotide polymorphisms (-800 G>A rs1800468, -509 C>T rs1800469, +10 T>C rs1800470, +25 G>C rs1800471, and +263 C>T rs1800472), showing that CD patients frequently express the TGF-β1 gene genotypes CT and TT at position -509, as compared to noninfected persons; similar results were observed with genotypes TC and CC at codon +10 of the TGF-β1 gene, leading to the conclusion that 509 C>T and +10 T>C TGF-β1 polymorphisms are associated with Chagas disease susceptibility. Studies in genetically different populations susceptible to CD will help to gather new insights and encourage the use of TGF-β as a CD biomarker.

## Introduction

### Chagas Disease: An Overview of Epidemiological, Economic, and Clinical Aspects

More than 100 years ago, *Trypanosoma cruzi* was identified as the etiological agent of Chagas disease (CD), which remains an important social and health problem in Brazil and Latin America, endemic in 21 countries according to the World Health Organization (WHO) (Araujo-Jorge et al., 2017; World Health Organization - WHO, 2021). CD is still considered a neglected tropical disease (NTD) and was inserted in the WHO road map for “ending the neglected to attain the sustainable development goals” as a major economic and public health problem in most Latin American countries (World Health Organization, 2021). Nowadays, around 6-7 million people are estimated to be infected by *T. cruzi* worldwide leading to a mortality near to 10,000 patients per year (World Health Organization - WHO, 2021) Approximately 22 million people live in areas at risk of contamination in Brazil (Pan American Health Organization, 2006), where the most recent Health Minister Bulletin (Brasil, 2021) indicates the current CD epidemiological profile: 1.36 - 3.2 million estimated infected persons for 2020, a mean of 4.663 deaths/year (2007-2017), with more than 320 new cases/year (2017-2019). Until 2020 only acute cases were mandatorily reported in but the new guidelines for notification of chronic cases (Brasil, 2021) and for treatment (CONITEC and Ministério da Saúde, 2018) are still challenges to be implemented as public health policies. No biomarkers are available to predict the risk of CD progression (CONITEC and Ministério da Saúde, 2018).

The net global amount used for medical care for individuals affected by CD is currently 24-73 billion dollars (Lee et al., 2013). Thus, the annual amount spent with CD results in an expense of US$ 4,660/person, and over the course of life an individual can generate US$27,684 in public expenses, including spraying insecticide to control vectors (Lee et al., 2013). The economic cost of CD is very similar or even higher compared to other diseases in the world, such as: rotavirus (US$ 2.0 billion), cervical cancer (US$ 4.7 billion), Lyme disease ($2.5 billion). These data reinforce the economic relevance for more attention and effort to CD control. The globalization process brought challenges and changes in the public health scenario in different countries (Gascon et al., 2010). Due to the migratory movement from the 1980s onwards CD became a concern in the developed world (Rodari et al., 2018). Therefore, we currently observe a large number of cases of the CD in non-endemic countries, such as Australia, Canada Spain and, US (Ribeiro et al., 2012; Forsyth et al., 2021), leading to a broad organization of affected person’s Associations (Brasil, 2021). About 59,000 to 108,000 individuals are estimated to be infected by *T. cruzi* on the European continent. Since CD is not transmitted by direct contact with the infected person, in non-endemic countries the relevant transmission mechanisms of *T. cruzi* are transfusion with infected blood, organ donation and congenital transmission (Martelli et al., 2017).

The natural history of CD includes two distinct and successive phases (CONITEC and Ministério da Saúde, 2018; Simões et al., 2018). The acute phase is characterized by a high parasite load in the bloodstream of the infected individual (parasitemia), a short period of time, starting between 6 to 10 days after infection and lasting on average 1-2 months in humans, with intense inflammatory response and parasitism of several cell types. Most acute cases through vector transmission are not reported, as the clinical symptoms are nonspecific, such as fever, malaise, headache, which are typical of many infections. In some cases, the presence of edema is observed at the site of the *T. cruzi* entry, the inoculation chagoma (skin inflammation causing an edematous swelling), which is called the Romaña’s sign when located on the eyelids (Araujo-Jorge et al., 2017). Severe acute Chagas disease is not common, but in cases of symptomatic patients at this stage, they include generalized adenopathy, hepatosplenomegaly, lymphadenopathy, meningoencephalitis, and myocarditis (CONITEC and Ministério da Saúde, 2018). However, serious symptoms such as mortality from encephalomyelitis or severe heart failure can occur, but these symptoms represent 5% of cases, in which most infected individuals are children from endemic regions (Ribeiro et al., 2012). Most acute cases in Brazil are currently related to oral transmission and present a different clinical scenario with a higher prevalence of symptomatic cases, acute myopericarditis and death (Pinto et al., 2008).

Untreated acute CD progresses to the chronic phase, which has two clinical forms: indeterminate and determined (Dias et al., 2016). In the indeterminate form, individuals have positive serology, but there are no symptoms or signs of target organ involvement confirmed by complementary tests such as X-ray and electrocardiogram. Most patients remain in the indeterminate chronic stage until the end of their lives, without developing symptoms of the determined chronic phase. However, after a period of 10-30 years, 15-30% of infected individuals evolve to the determined forms of the disease, associated with tissue damage that includes cardiac, digestive, and mixed forms (Dias et al., 2016).

From the patients who move on to the chronic phase, 20-30% develop cardiac form, up to 10% exhibit digestive form, and it is still possible to identify mixed form (cardiac and digestive), in less than 5% (Gascón et al., 2007). The chronic CD cardiac form (CCC) is the most important cause of morbidity and mortality among people affected by CD, also resulting in a significant medical and social impact (Rassi et al., 2010). The most important CCC manifestations are arrhythmias, including sudden cardiac arrest, heart failure, and stroke. However, many patients may present stroke or sudden cardiac arrest as the first clinical presentation of the cardiac form. Regarding the digestive form, the most important presentations are megaesophagus and megacolon (Gascón et al., 2007; Henao-Martínez et al., 2012; Dias et al., 2016).

A variety of structural changes in the cardiovascular system have been described in patients with CCC (Rassi et al., 2017). The main change is observed in the reparative and fibrotic process, with a diffuse and dense accumulation of interstitial collagen that involves individual fibers or even an entire group of fibers. Thus, heart fibrosis is the most important histopathological outcome in CCC, both in humans and in experimental models (Rassi et al., 2010). Fibrosis is defined as an excessive deposition of extracellular matrix components in organs and tissues, because of the fibroblasts´ proliferation and activation, triggered by pro-inflammatory cytokines produced by cells of the innate and adaptive immune system (Wick et al., 2013). Basically, this is a process where the damaged tissue is replaced by connective tissue resulting in remodeling and in the functional impairment of the organ (Pohlers et al., 2009). The fibrosis pattern varies from focal to diffusely distributed fibrosis (Simões et al., 2018). Furthermore, the fibrotic reaction molded by the connective tissue after the inflammatory response is mainly characterized by an increase of extracellular matrix components production and by proliferation, migration, and accumulation of mesenchymal cells. When the inflammatory process ceases, the fibrotic process is sustained, and the installed fibrosis compromises the correct functionality of damaged tissues and organs (Pohlers et al., 2009). Some molecules influence these processes but transforming growth factor beta cytokine (TGF-β) plays a key role in this process by inducing the synthesis of extracellular matrix components and decreasing its degradation and turn-over (Leask, 2004).

### Transforming Growth Factor Beta and Its Intracellular Signaling Pathways

In the early 1980s, it became evident that cell growth was controlled by different polypeptides and hormones (Sporn and Todaro, 1980) and a new polypeptide called TGF-β was identified in neoplastic cells (Roberts et al., 1981; Eisinger et al., 1988): a growth factor that induced proliferative phenotype in fibroblasts and collagen production *in vivo* and *in vitro*. Shortly after its discovery, a dual role of this cytokine was described, also showing the inhibition of cell proliferation. In these cells, TGF-β acted synergistically with another platelet-derived growth factor and inhibited colony formation (Roberts et al., 1985). TGF-β is a homodimeric protein that is part of the TGF-β superfamily and is found in most eukaryotic organisms, including *C. elegans*, *Drosophila*, *Xenopus*, rats, and humans (Harada et al., 2015). It is expressed in all cell types and in almost all developmental stages of organisms, playing an important role in the regulation of various biological and cellular responses, including cell proliferation and differentiation, extracellular matrix production, embryonic development, epithelial cell growth, carcinogenesis, and apoptosis (Massagué, 2012). In mammalian cells, there are three TGF-β subtypes: 1, 2 and 3. These isoforms are well characterized as small (25 kDa) homodimeric secreted proteins (Wilson, 2021) that are encoded by distinct genes located on different chromosomes. These molecules present significant homology (80%) and are 100% conserved across species, consisting of two monomers with a cysteine core linked by a disulfide bond (Huang et al., 2014). Although the three-dimensional structures of the isoforms are similar, intrinsic differences contribute to the activity and existence of each of these isoforms *in vivo* (Huang et al., 2014).

**Figure 1:**
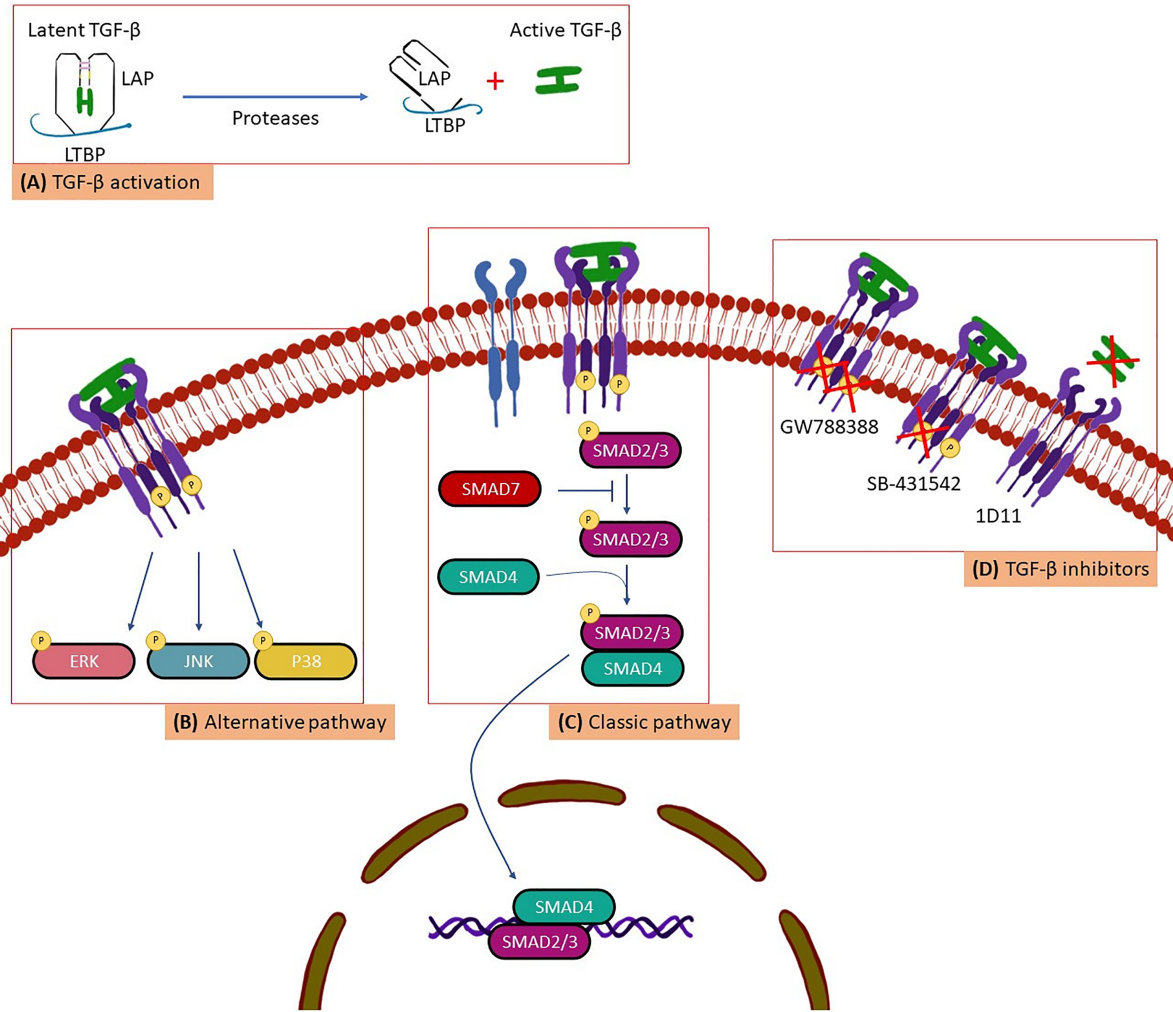
A scheme for **(A)** TGF-β activation, **(B)** alternative pathway, **(C)** Classic pathway and, **(D)** TGF-β inhibitors.

TGF-β synthesis is widespread in all cell types (Saharinen et al., 1996). However, due to its pleiotropic activity TGF-β is under strict control in organisms and is secreted in its latent, biologically inactive form (**Figure 1**). This latent inactive TGF-β is synthesized in a dimeric precursor form associated with the latency-associated protein (LAP), forming the small latent complex (Saharinen et al., 1996; Massagué and Chen, 2000). To perform its biological activities TGF-β needs to be activated to further interact with type I (TβRI), type II (TβRII) and type III (TβRIII) surface receptors, identified in virtually all cell types (Massagué and Gomis, 2006). Thus, activated TGF-β is recognized by its surface receptors following a time sequence (**Figure 1**): (i) initially, TβRIII acts by modulating the interaction between TGF-β with the appropriate signaling receptors (TβRI and TβRII). Therefore, TβRIII binds to TGF-β and directs it to TβRII; (ii) the binding of TGF-β to TβRII induces the dimerization and formation of a TβRI and TβRII complex, in which TβRII phosphorylates the serine/threonine domain of the TβRI resulting in the activation of an intracellular signaling cascade (Massagué, 1998; Massagué and Chen, 2000; Massagué and Gomis, 2006; Massague, 2008; Massagué, 2012) (**Figure 1**) Two types of signaling pathway triggered by active TGF-β have been described (**Figure 1**): the classic pathway, when the TβRI phosphorylates and activates the SMADs proteins (considered as important markers for the activation of the TGF-β signaling pathway) and the alternative pathway, when TβRI phosphorylates and activates other proteins such as: mitogen-activated protein kinases (ERK), C-Jun N-terminal kinase (JNK) and p38 mitogen-activated protein kinases (Massague and Wotton, 2000; Massagué and Gomis, 2006). This process results in the recruitment of transcriptional cofactors or corepressors that lead to transcriptional activation or repression of TGF-β responsive genes (Shi and Massague, 2003).

### TGF-β and Chagas Disease

The first study involving the role of TGF-β in the development of CD was carried out in 1991, observing that, when peritoneal macrophages from mice and humans were treated with TGF-β, the trypanocide capacity of IFN-γ was inhibited (Silva et al., 1991). Furthermore, the treatment of human macrophages with TGF-β resulted in increased parasite replication. The effect of TGF-β on *T. cruzi* infection *in vivo* was also investigated. Mice infected by *T. cruzi* and treated with TGF-β developed higher parasitemia with decreased survival. The authors then proposed that TGF-β could play an important role in the regulation of the *T. cruzi* infection (Silva et al., 1991).

In 1996, Zhang and Tarleton (1996) observed increased TGF-β levels in inflammatory and regulatory cells in the spleen from different *in vivo* experimental models, at the beginning of the acute phase of *T. cruzi* infection. They also described that the peak of TGF-β production in the spleen of infected animals was concomitant with the peak of parasitemia in the three experimental models analyzed (Zhang and Tartelon, 1996). In addition, Zhang and Tarleton observed many TGF-β-producing cells in the heart tissue of infected animals during acute phase and persisting through the chronic phase (Zhang and Tartelon, 1996). A study developed with *T. cruzi*-infected primates also showed that TGF-β was produced in the first week of the acute phase and was constantly and systemically expressed during the chronic phase of the infection (Samudio et al., 1999). The gene expression of TGF-β increased in the heart of primates from two weeks after infection, remaining increased up to 10 years after infection (Samudio et al., 1999).

In 2002, Araújo-Jorge et al. (Araujo et al., 2002) observed that CCC patients had on average 10-20 times higher levels of circulating TGF-β1 when compared to healthy individuals (Araujo et al., 2002). These same patients were re-evaluated, and we demonstrated the predictive value of TGF-β1 as a biomarker of clinical progression in Chagas disease: patients in the early stages of the chronic phase, who presented high levels of circulating TGF-β1, evolved with worse prognosis after 10 years of follow-up (Saraiva et al., 2013). *T. cruzi* infection in different cell lines (human fibroblasts and epithelial cells) also resulted in a significant increase in the production of TGF-β in the supernatant of these cultures (Martello et al., 2013). An intriguing data was obtained in a further study that described low levels of TGF in stages C and D (Curvo et al., 2018). These results were justified because of different treatment schemes with carvedilol or spironolactone, since these drugs improved the survival of heart failure, but decreased TGF-β transcription.

We also demonstrated another important TGF-β role in Chagas disease: its involvement in cardiac tissue homeostasis, acting as a regulator of cell proliferation and death, extracellular matrix remodeling, electric coupling, and angiogenesis (Araujo-Jorge et al., 2012). Therefore, this cytokine is a key molecule to be studied in infectious diseases that damage the heart tissue, such as CD. Immunohistochemical analyzes of cardiac biopsies from patients with CCC presenting moderate or severe cardiac dysfunction showed intense staining for fibronectin in the extracellular matrix, as well as the presence of phosphorylated Smad2 (Araujo et al., 2002).

In addition to participating in the different processes already described in the progression of the CD, studies have shown that TGF-β also acts in the control of the different stages of *T. cruzi* life cycle (Waghabi et al., 2005; Waghabi et al., 2007). Waghabi et al. (2005) demonstrated that *T. cruzi* directly activates latent TGF-β, as a necessary strategy for host cell invasion. Ten years later, it was described that this activation is performed by a cysteine peptidase produced by *T. cruzi*, called cruzipain (Ferrão et al., 2015). These results represented the first example in the literature of a new mechanism by which a protozoan uses a host cell molecule, TGF-β, to control its own intracellular life cycle (Waghabi et al., 2005).

In 2016, we analyzed the kinetics of the TGF-β signaling pathway during the acute phase of experimental CD, observing that the *T. cruzi* infection: (i) significantly increases the expression of receptors: TβRI and TβRII; (ii) stimulates the phosphorylation of both the classical signaling pathway proteins (Smad2/3) and the alternative one (JNK, p38, ERK); (iii) significantly increases the TGF-β1 mRNA levels; (iv) leads to a high expression of TGF-β-responsive proteins: CTGF and fibronectin and; (v) with increased collagen deposition (Ferreira et al., 2016). Once again, these data confirm the main role of TGF-β in the development and maintenance of cardiac damage in response to *T. cruzi* infection (Ferreira et al., 2016). Silva et al. (2019) also determined TGF-β regulatory mechanisms in CD. This study in *Trypanosoma cruzi*-infected cardiomyocytes and cardiac fibroblasts demonstrated that p38 and c-Jun pathways could participate in regulatory process of fibrosis mediated by TGF-β (Silva et al., 2019). A recent *in-silico* study showed the prediction and verification of nine potential genes that were strongly associated with the virulence mechanisms of *T. cruzi* and the host immune response. One of these target genes was a member of the Smad5 family, a protein involved in the classical TGF-β signaling pathway (Ballinas-Verdugo et al., 2021). Other *in-silico* study described that some known and novel PIWI-interacting RNA (piRNAs) from the host could be dysregulated and could target and potentially regulate the expression of genes including TGF-β and two other piRNAs target genes, with one degree of interaction to TGF-β1. However, the role of these piRNAs in the CD pathogenesis remains unknown (Rayford et al., 2020).

CD susceptibility and its clinical manifestations could be influenced by genetic factors of the host (Ayo et al., 2013). A review article updated the current knowledge of the genetic basis of Chagas disease. Acosta-Herrera at el. (2019) concluded that more than 50 polymorphisms are associated with the susceptibility to *T. cruzi* infection and to the chronic manifestation of Chagas disease (Acosta-Herrera et al., 2019). In 2009, a study carried out in Peru and Colombia identified the association of TGF-β1 gene polymorphisms with susceptibility to the development of CD. Calzada et al. (2009) observed that the T allele at codon 10, which is associated with low production of TGF-β1, was found more frequently in healthy individuals than in cardiac patients. Conversely, the frequency of the C allele, which is associated with high production of TGF-β1, was higher in the group of infected individuals, indicating that this allele is a risk factor for susceptibility to the development of the disease (Calzada et al., 2009).

In 2018, we evaluated the importance of the TGF-β1 polymorphism in Brazilian patients in the chronic phase, including the indeterminate form and the different stages of the cardiac form. We also correlated the expression of different TGF-β1 alleles with the susceptibility of CD development. We demonstrated that two TGF-β1 polymorphisms, -509 C>T (rs1800469) and codon 10 T>C (rs1800470), are associated with the susceptibility to the development of the disease in Brazilian cohort (Ferreira et al., 2018). Thus, TGF-β1 is a potential serological marker with predictive value in the clinic for patients in the early stages of the disease. Considering the extensive involvement of TGF-β in the development of infectious and genetic diseases, in cell proliferation and differentiation, extracellular matrix production and consequent fibrosis, inhibition of TGF-β activity could be a possibility for treatment or even cure of some disease and biological and cellular processes. In other infections such as HIV (Jiang et al., 2021) and COVID-19 (Carlson et al., 2020) the important role of TGF-β in the remodeling of the extracellular matrix is also proposed, contributing to fibrosis. TGF-β involvement in various diseases increased the efforts for the development of compounds that inhibit the activity and signaling pathway of this molecule. Thus, our group has intensively evaluated pre-clinically the therapeutic action of some pharmacological compounds that inhibit the activity of TGF-β in CD, such as SB-431542, GW788388 and 1D11 (Araujo-Jorge et al., 2012) (**Figure 1**).

In 2007, Waghabi et al. (2007) evaluated the role of SB-431542 (TβRI inhibitor) in an *in vitro* infection model. As shown in the **Table 1**, we observed that the compound: (i) inhibits the activation of the TGF-β pathway induced by the presence of the parasite in epithelial cells and cardiomyocytes; (ii) reduces *T. cruzi* invasion in cardiomyocyte; (iii) decreases the number of parasites per infected cell; (iv) impairs *T. cruzi* differentiation, and release of trypomastigote forms, and (v) induces the death of intracellular parasites. We also evaluated the effect of SB-431542 on Gap junction proteins connexin-43 (Cx-43) finding a reduction and disorganization profile in the expression of Cx-43 (Waghabi et al., 2009a). These changes contribute to the abnormal conduction of the electrical impulse, which was reserved after treatment with SB-431542, resulting in an increased expression of Cx-43 and reestablishment of the organization of its plaque structure in cardiomyocytes. Thus, for the first time in the literature, we demonstrated in animal models that inhibition of the TGF-β pathway could be tested as a possibility for the treatment of CD.

**Table 1:** Pre-clinical and clinical approaches implicating TGF-β in modulation of pathology in Chagas disease.

Year	Authors	Ref	Subject	Effects/Results	Host//model
1991	Silva et al.	(Massague and Wotton, 2000)	TGF-β treatment *in vitro* and *in vivo*	*T. cruzi* infected mice treated with TGF-β developed high parasitemia with decreased survival	Mice
1996	Zhang and Tarleton	(Shi and Massague, 2003)	TGF-β treatment *in vivo*	Increased TGF-β levels at the beginning of the acute phase of Chagas disease	Mice
1996	Zhang and Tarleton	(Silva et al., 1991)	TGF-β treatment *in vivo*	Large number of TGF-β-producing cells in the heart tissue during acute infection	Mice
1999	Samudio et al.	(Zhang and Tarleton, 1996)	TGF-β treatment *in vivo*	TGF-β is produced in the first week of the acute phase and is constantly expressed	Monkeys
2013	Martello et al.	(Araujo et al., 2002)	TGF-β treatment *in vitro*	Increased production of TGF-β in the infected cultures	Cells - *in vitro*
2016	Ferreira et al.	(Waghabi et al., 2005)	TGF-β treatment *in vivo*	*T. cruzi* infection increased the TGF-β1 mRNA levels	Mice
2002	Araujo-Jorge et al.	(Zhang and Tartelon, 1996)	TGF-β status	High levels of circulating TGF-β1 in chronic patients	Patients
2009	Calzada et al.	(Ballinas-Verdugo et al., 2021)	TGF-β status	TGF-β1 polymorphisms are associated with the susceptibility to the development of the disease	Patients
2018	Ferreira et al.	(Rayford et al., 2020)			
2007	Waghabi et al.	(Curvo et al., 2018)	TGF-β inhibition *in vitro*	Induces the presence of the parasite in epithelial cells and cardiomyocytes	Cells - *in vitro*
2007	Waghabi et al.	(Curvo et al., 2018)	TGF-β inhibition *in vitro*	Reduces cardiomyocyte invasion and infection	Cells - *in vitro*
2007	Waghabi et al.	(Curvo et al., 2018)	TGF-β inhibition *in vitro*	Decreases the number of parasites per infected cell	Cells - *in vitro*
2007	Waghabi et al.	(Curvo et al., 2018)	TGF-β inhibition *in vitro*	Reduces the differentiation and release of trypomastigote forms	Cells - *in vitro*
2007	Waghabi et al.	(Curvo et al., 2018)	TGF-β inhibition *in vitro*	Induces the death of intracellular parasites	Cells - *in vitro*
2009	Waghabi et al.	(Ferreira et al., 2018)	TGF-β inhibition *in vivo*	Declines mortality	Mice
2012	Oliveira et al.	(Jiang et al., 2021)			
+2009	Waghabi et al.	(Ferreira et al., 2018)	TGF-β inhibition *in vivo*	Decreases parasitemia	Mice
2012	Oliveira et al.	(Jiang et al., 2021)			
2009	Waghabi et al.	(Calzada et al., 2009)	TGF-β inhibition *in vivo*	Improves the electrocardiographic profile	Mice
2009	Waghabi et al.	(Calzada et al., 2009)	TGF-β inhibition *in vivo*	Reduces the enzymatic tissue damage biomarkers	Mice
2009	Waghabi et al.	(Calzada et al., 2009)	TGF-β inhibition *in vivo*	Prevines aggressive damage	Mice
2012	Oliveira et al.	(Jiang et al., 2021)			
2019	Ferreira et al.	(Carlson et al., 2020)			
2012	Oliveira et al.	(Jiang et al., 2021)	TGF-β inhibition *in vivo*	Maintains cardiac electrical conduction and baseline of Cx43 expression	Mice
2019	Ferreira et al.	(Carlson et al., 2020)			
2012	Oliveira et al.	(Jiang et al., 2021)	TGF-β inhibition *in vivo*	Decreases cardiac fibrosis	Mice
2019	Ferreira et al.	(Carlson et al., 2020)			
2019	Ferreira et al.	(Carlson et al., 2020)	TGF-β inhibition *in vivo*	Decreases circulating TGF-β levels	Mice
2019	Ferreira et al.	(Carlson et al., 2020)	TGF-β inhibition *in vivo*	Reduces the expression and activities of SMAD2/3 proteins	Mice
2019	Ferreira et al.	(Carlson et al., 2020)	TGF-β inhibition *in vivo*	Increases the activation of MMP2 and MMP9	Mice
2019	Ferreira et al.	(Carlson et al., 2020)	TGF-β inhibition *in vivo*	Decreases of the protein expression of TIMP1/2/4	Mice
2019	Ferreira et al.	(Carlson et al., 2020)	TGF-β inhibition *in vivo*	Recovers the transcription of markers of cardiac regeneration	Mice

In 2009, Waghabi et al. (2009) demonstrated that male Swiss mice infected with the *T. cruzi* Y strain and treated with SB-431542: (i) reduces mice mortality; (ii) decrease in parasitemia; (iii) improve the electrocardiographic profile; and (iv) reduces the enzymatic tissue damage biomarkers (aspartate aminotransferase and creatine kinase). Thus, the inhibition of the TGF-β signaling pathway *in vivo* significantly attenuate the infection, preventing aggressive damage to the heart of infected animals (Waghabi et al., 2009b). The therapeutic activity of another TGF-β pathway inhibitor was also evaluated in an acute infection model of CD: male Swiss mice were infected with the *T. cruzi* Y strain and treated with GW788388. The compound was administered at the beginning of the infection and reduced parasitemia, increased animal survival, maintained cardiac electrical conduction and baseline of Cx43 expression. In addition, administration of GW788388 at the end of the acute phase increased animal survival and decreased cardiac fibrosis. These results reaffirm that inhibition of the TGF-β signaling pathway could be considered an important alternative strategy for the treatment of cardiomyopathy developed in CD (Oliveira et al., 2012). Another outstanding study established a therapeutic approach for controlling *T. cruzi*-driven fibroblast differentiation by poly [ADP-ribose] polymerase 1 inhibitors through modulation of the profibrotic macrophages signaling of the activator protein 1-MMP9 -TGF-pathway, thus controlling chronic fibrosis in CD by indirect inhibition of TGF-β’s functions (Choudhuri and Garg, 2020).

More recently, we also investigated the effect of GW788388 in a chronic CD experimental model (Ferreira et al., 2019). Ferreira et al. (2019) observed that treatment with GW788388 had a relevant therapeutic effect on the cardiac tissue of *T. cruzi* infected mice, resulting in: (1) decreased circulating TGF-β levels; (2) improved the electrocardiographic profile: decrease of the PR and QTc intervals, increase in heart rate, reversal of sinus arrhythmia and reversal of disturbances in atrial and atrioventricular conduction; (3) reversed the altered formation of intercellular plaques enriched with Cx-43; (4) decreased fibrosis in cardiac tissue; (5) decreased of the expression and activities of SMAD2/3 proteins, important proteins involved in TGF-β intracellular signaling pathway; (6) increased of the activity of MMP9, an enzyme important in the process of degradation of extracellular matrix proteins to decrease fibrosis; (7) decreased the protein expression of TIMP1/2/4, which are inhibitors of the activity of MMPs; and (8) recovered the transcription of GATA-6 and Tbox-5 genes, important markers of cardiac regeneration (Ferreira et al., 2019). Thus, the therapeutic effects of the TGF-β signaling pathway inhibition are promising and suggest a new possibility for the treatment of cardiac fibrosis in the chronic phase of CD.

Some studies evaluated the inhibition of the TGFβ-signaling pathway, and some molecules are under investigation in clinical trials for other diseases. AVID200, a potent TGF-beta 1 and 3 inhibitor, entered clinical trials phase I for the treatment of patients with advanced solid tumors. Weill Medical College of Cornell University New York, United States, is now recruiting patients for the investigation of safety and feasibility of Vactosertib, a small TGF-β type I receptor inhibitor molecule for the treatment of anemic chronic myeloproliferative neoplasms patients. Translation of the *in vitro* and *in vivo* result of TGF studies on Chagas disease development to patients into clinical trials is challenging but, as well as to other disease, could represent a new therapeutic strategy to be assessed.

## Concluding Remarks

Chagas disease affects approximately 8 million people in Latin America, and its prevalence in non-endemic countries has increased due to increasing international migration and non-vector transmission routes. The present drugs available for the treatment of CD in acute and chronic phases are first-generation trypanocidal drugs (benznidazol and nifurtimox) and only few new drugs are under evaluation in clinical trials, with no focus on cardiac physiopathological mechanisms of CD. Therefore, research to identify new treatments for CD is needed. To decrease the impact of CD on the population and the public health budget, governments must invest in treatment and health promotion policies as well as on innovation for new treatment strategies. The role of TGF-β as an inducer of several pathological processes leading to development and maintenance of cardiac damage in CD transforms this cytokine in a relevant target for the advancement of therapies for CCC patients. The therapeutic effects of inhibiting the TGF-β signaling pathway, *in vitro* and *in vivo*, are well described and promising. Thus, evaluation of the effect of the TGF-β signaling pathway inhibitors could be considered as: (i) a new strategy for the treatment of cardiac fibrosis in CD; (ii) a suggestion of effective and specific treatment for CCC. After 20-years of work in this line of research, we consider these conclusions as an important outcome.

## Author Contributions

All authors contributed to the concept and execution of the experiments that generated the ideas presented in this review. RF and TA-J wrote the original draft and all the co-authors reviewed and complemented the text. All authors contributed to the article and approved the submitted version.

## Funding

We received funding by the Department of the Industrial Complex and Innovation in Health from the Brazilian Health Ministry (IOC Fiotec IOC-001-LIV-11-2-1; 25380.001603/2017-89; www.saude.gov.br/sctie), the governmental agencies Conselho Nacional de Desenvolvimento Científico e Tecnológico (CNPq 309545/2014-5; 313011/2018-4, 159947/2018-9);, Fundação de Amparo à Pesquisa no Rio de Janeiro Carlos Chagas Filho (FAPERJ E26/201.838/2017; 201.983/2020; www.faperj.br); and Oswaldo Cruz Institute (IOC/Fiocruz; www.ioc.fiocruz.br).

## Conflict of Interest

The authors declare that the research was conducted in the absence of any commercial or financial relationships that could be construed as a potential conflict of interest.

## Publisher’s Note

All claims expressed in this article are solely those of the authors and do not necessarily represent those of their affiliated organizations, or those of the publisher, the editors and the reviewers. Any product that may be evaluated in this article, or claim that may be made by its manufacturer, is not guaranteed or endorsed by the publisher.
